# A Soft Resistive Sensor with a Semicircular Cross-Sectional Channel for Soft Cardiac Catheter Ablation

**DOI:** 10.3390/s21124130

**Published:** 2021-06-16

**Authors:** Eric Rasmussen, Daniel Guo, Vybhav Murthy, Rachit Mishra, Cameron Riviere, Carmel Majidi

**Affiliations:** 1 Surgical Mechatronics Laboratory, Robotics Institute, Carnegie Mellon University, Pittsburgh, PA 15213, USA; erasmuss@andrew.cmu.edu (E.R.); criviere@andrew.cmu.edu (C.R.); 2 Department of Mechanical Engineering, Carnegie Mellon University, Pittsburgh, PA 15213, USA; dguo2@andrew.cmu.edu (D.G.); vybhavm@andrew.cmu.edu (V.M.); rachitm@andrew.cmu.edu (R.M.); 3 Soft Machines Laboratory, Department of Mechanical Engineering, Carnegie Mellon University, Pittsburgh, PA 15213, USA

**Keywords:** soft sensing, FEA modeling, cardiac catheter ablation, neo-Hookean model

## Abstract

The field of soft robotics has attracted the interest of the medical community due to the ability of soft elastic materials to traverse the abnormal environment of the human body. However, sensing in soft robotics has been challenging due to the sensitivity of soft sensors to various loading conditions and the nonlinear signal responses that can arise under extreme loads. Ideally, soft sensors should provide a linear response under a specific loading condition and provide a different response for other loading directions. With these specifications in mind, our team created a soft elastomeric sensor designed to provide force feedback during cardiac catheter ablation surgery. Analytical and computational methods were explored to define a relationship between resistance and applied force for a semicircular, liquid metal filled channel in the soft elastomeric sensor. Pouillet’s Law is utilized to calculate the resistance based on the change in cross-sectional area resulting from various applied pressures. FEA simulations were created to simulate the deformation of the sensor under various loads. To confirm the validity of these simulations, the elastomer was modeled as a neo-Hookean material and the liquid metal was modeled as an incompressible fluid with negligible shear modulus under uniaxial compression. Results show a linearly proportional relationship between the resistance of the sensor and the application of a uniaxial force. Altering the direction of applied force results in a quadratic relationship between total resistance and the magnitude of force.

## 1. Introduction

Over the course of the previous few decades, medical practitioners have strived to move towards minimally invasive procedures to further protect their patients [1]. Minimally invasive surgery (MIS) can be defined as a style of surgery in which surgeons use various techniques to cause the least amount of damage to the body without compromising the intended outcome of the procedure [2]. Due to decreased amount of damage on the body during MIS, patients experience less pain, less scarring, and have shorter hospital stays among other positive outcomes [2,3,4]. In more recent years, robotic MIS has become a more popular option, as human error such as natural tremor can be mitigated [4,5,6]. However, current robotic configurations utilize rigid structures and stiff materials to perform surgery, making it difficult for the devices to navigate through the abnormal crevices of the body [6]. Other complications include 3D mapping error of the surgical environment, electrical sparking, and burnt pieces of the apparatus being lost inside the patient [7].

Soft robotics have become increasingly appealing for robotic minimally invasive surgery (RMIS) due to their improved ability to traverse the inside of the human body compared to traditional rigid robotics. In contrast to conventional machines, soft robots are created out of flexible material, giving them the ability to adapt and conform to their external environment [6,8,9]. Through the use of conductive ink, liquid metal, and other aqueous conductors, flexible circuitry can be integrated into these soft robotic systems [7,10,11,12], creating the ability to manufacture soft pressure, strain, and positional sensors that can be utilized in tandem with other tools during surgery [13,14,15]. However, processing the signals produced from these sensors can be challenging for a variety of reasons. Liquid metal sensors typically exhibit a nonlinear response to mechanical loading and can produce differing results over time due to creep when subjected to cyclic loading [6,16,17]. Hysteresis caused by internal energy dissipation between mechanical loading and unloading can also produce unequal sensor responses when the same load is applied, dependent on the direction of load [16,17]. 

**Figure 1:**
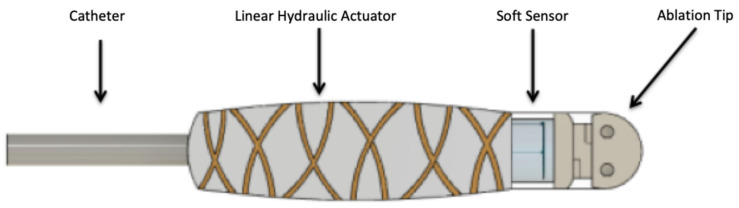
A 2D rendered image of a soft linear actuator intended for cardiac catheter ablation described in [18]. The soft device consists of a linear hydraulic actuator that can be pressurized using inert saline solution pumped in through the catheter line. The fibers wrapped around the actuator allow for only one degree of freedom when pressured, so the device can extend and retract perpendicular to the heart wall. The soft sensor that will be expanded on in this paper will be housed in line with the ablation tip in order to accurately output the compressive force experienced by the ablation tip during contact with cardiac tissue. The electrodes and wiring normally associated with radio-frequency ablation is not shown in this theoretical portrayal.

**Figure 2:**
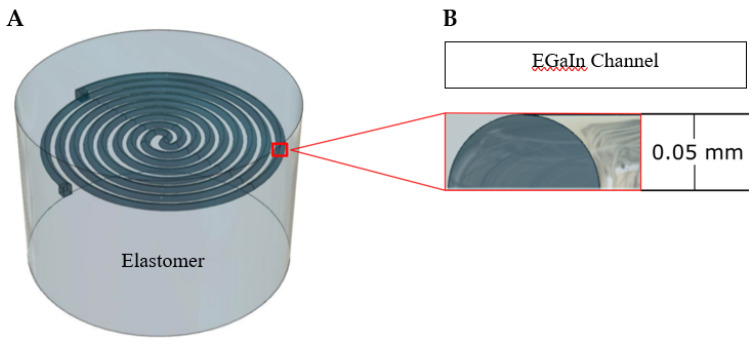
(**A**) A 3D rendered image of the soft microsensor design based on the novel fabrication method created by Parekh et al. [19] and spiral design featured in [15]. The sensor exhibits a semicircular channel cross section due to the resulting geometry of 3D printing liquid metal onto cured elastomer. (**B**) The semicircular channel is embedded 0.5 mm beneath the surface of the elastomeric shell of the sensor. The channel forms a spiral structure to maximize the length and resolution of the sensor.

Recently, our research team designed a soft actuator and sensor for cardiac catheter ablation for soft RMIS (Figure 1). The device is intended to augment normal cardiac catheter ablation with the addition of dynamic force control to treat atrial and ventricular fibrillation [18]. Utilizing a McKibben muscle to perform a linear motion, the device is intended to apply a consistent known force via the soft sensor and a force control algorithm. This paper will largely focus on the creation and characterization of a soft, resistive strain sensor that can be utilized within catheter device as well as the electrically stimulated ventricles of the heart (Figure 2). Parekh et al. described a novel fabrication method for liquid metal-filled microchannels with a semicircular cross section via 3D liquid metal printing [19]. The accuracy and consistency of this simple process leads to high reproducibility when creating liquid metal microchannels. A spiral pattern featured in [15] was used to maximize the liquid channel length of the sensor, in turn increasing its sensitivity. With this design in mind, our team developed finite element analysis (FEA) simulations to fully characterize a soft microresistive strain sensor with a semicircular cross section. A neo-Hookean analytical model was used to confirm the validity of the results obtained by the FEA simulations. Based on Pouillet’s Law, we expect a linear, proportional correlation between the resistance and force applied. The directionality of force applied and its effect on resistance is also investigated.

Cardiac catheter ablation procedural success rate is heavily dependent on the tool-to-tissue contact force [20,21,22]. While manual controlled catheters may use contact force ranges between 10 and 40 g (0.1–0.4 N) [23] the recommended target area associated with higher successful rates is a contact force of 10–20 g (0.1–0.2 N) [21,22]. Based on these clinical standards, our sensor will be characterized between 0.1 and 0.4 N to cover the full range of accepted contact force values for successful cardiac ablation. 

## 2. Methodology

### 2.1. Design Overview

Our sensor consists of a circular elastomeric frame with a radius of 1.5 mm. The total height of the sensor is 2.0 mm with the semicircular channel resting at the 1.5 mm mark, half a millimeter below the surface. A schematic of the design was created in Fusion 360 3D modeling software and can be seen in Figure 1 and Figure 2. The channel spans a total of 37.4 mm, shaped in a fashion to maximize its length, as first demonstrated in [15]. According to Pouillet’s Law, a larger channel length correlates to a finer change-in-resistance resolution when the channel is deformed. The radius of the semicircular channel is 50 microns. Ecoflex-0030 is typically used as the soft silicone matrix material due to its low durometer and elastic modulus. The liquid metal eutectic gallium indium (EGaIn) is used for its high electrical conductivity, low viscosity, and structural integrity that arises from its passivating oxide layer [12,19]. Both materials will be modeled in our computational analysis. 

While this article focuses on the computational characterization of the sensor, it is based off of an already proven fabrication method [19]. To fabricate this model, EGaIn can be printed in the shape of the channel onto a cured elastomer substrate. Uncured elastomer of the same chemical composition as the base can be poured on the liquid metal without deformation of the channel shape due to the aforementioned passivating oxide layer. This oxide layer is also the reason the 3D liquid metal reservoirs can be created, as the structural integrity gained by the oxide layer allows for 3D pillars of EGaIn to be extruded. Wires can be inserted into the reservoirs prior to when the top layer has fully cured, or before the final layer is poured. Once the final layer of elastomer is cured, the shell of the sensor can be removed from the excess elastomer. 

### 2.2. Finite Element Simulation

To determine the change in resistance through finite element analysis, we simulated a parametric sweep of force (0 N–0.4 N, with a step size of 0.1 N) applied uniformly across the entire surface of the sensor. An increase in force across the sensor will increase the deformation of the soft elastomer, and in turn, deform the microchannel. Assuming that EGaIn and the elastomer are incompressible, it can be assumed that the liquid metal channel will retain a constant volume, despite deformation via pressure. By comparing the initial and post deformation length of the channel, the cross-sectional area of the channel at each mesh point can be determined using the constant volume assumption. 

The Cartesian coordinates for the initial and post deformation mesh points along the channel were recorded. The three-dimensional distance formula in Equation (1) was used to calculate the length of each line segment along the channel pre and post deformation.

(1) $$d=\sqrt{{\left(x_{1}-x_{2}\right)}^{2}+{\left(y_{1}-y_{2}\right)}^{2}+{\left(z_{1}-z_{2}\right)}^{2}}$$

Here, *d* is the distance between two points, and *x_i_*, *y_i_*, *z_i_* are the Cartesian coordinates where the index i corresponds to the points 1 and 2. Point 1 and point 2 refer to the same point pre and post deformation, respectively. N = 324 equidistant points are used to make up 323 line segments along the 37.4 mm channel. The sum of the initial line segments was cross-referenced with the 3D model to confirm an accurate mesh representation. There was a 99.7% agreement in length between the mesh representation and 3D CAD model. The resistance of each line segment can be calculated using Pouillet’s Law seen in Equation (2):

(2) $$R=\frac{\rho L}{A}$$

where *R* represents the resistance of the line segment, ρ is the initial resistivity of a uniform material (EGaIn), *L* is the length of the channel segment, and *A* represents the cross-sectional area of the segment. The sum of the resistances across all line segments is equivalent to the total resistance across the sensor. The change in resistance as a function of force was found using the initial resistance of the 37.4 mm channel and using this method to find the total resistances of each force applied in the sweep. 

**Figure 3:**
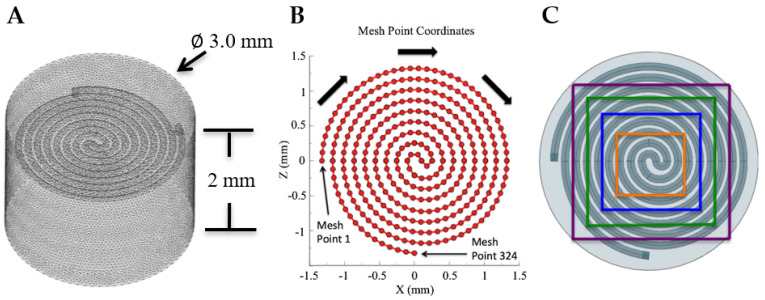
(**A**) A 0.05 mm triangular mesh size was used to characterize the soft sensor using finite element analysis via Matlab’s FEAtool application. (**B**) A 2D graphical depiction of the mesh point locations that were used to model the deformation of the liquid metal channel. The first recorded point begins at the leftmost point of the spiral structure and follows the arrows around the structure until it reaches the final mesh point at the bottommost point of the channel. (**C**) Areas outlined by the color-coded squares represent the area under compression during pressure directionality testing.

A virtual mesh was created using the application Gmsh. A triangle mesh was used with the maximum mesh size being 0.05 mm. Figure 3 depicts the 3D mesh configuration of the sensor. Matlab’s FEAtool application was used to run linear elastic deformation simulations on the mesh imported from Gmsh. Linear elastic deformation has been used to model elastomeric materials with small amounts of strains in the past [15]. The Poisson’s ratio of the material was set to 0.499, while the density and elastic modulus of the material were set to 1070 kg/m^3^ and 125 KPA, respectively, based on the innate properties of EcoFlex 0030.

### 2.3. Pressure Directionality 

The resistance as a function of surface area deformation was also investigated. A constant 0.4 Newton force was applied centrally as a parametric sweep of the surface area under deformation was conducted. The sweep began with the deformation of a 0.8 by 0.8 mm square surface area and ended with a 2 by 2 mm square surface area. The same procedure previously mentioned was used to compute the total resistance of each simulation. 

Finally, the resistance as a function of lateral loading pressure was examined. Starting with the full surface area of the sensor, the surface area was decreased laterally along the x-axis while keeping a constant 0.4 force applied. The results of these pressure sweeps allow the ability to predict the resistance output under various partial loads and provide insight on the importance of accounting for directionality in soft sensing. 

### 2.4. Analytical Model—Neo-Hookean Material

The neo-Hookean model is a simple method to model hyperelastic materials, similar to that of Hooke’s Law [24]. It is often used to describe the nonlinear stress–strain curves of both compressible and incompressible materials. Rubber has been considered an incompressible material in modeling due to its bulk modulus being many magnitudes higher than its shear and Young’s moduli [24,25,26]. The principal stretch ratios of an incompressible neo-Hookean material can be described in Equation (3) below:

(3) $$\lambda_{1}\lambda_{2}\lambda_{3}=1$$

The principal stretch ratios of a rectangular channel will be used to describe our model and are as follows:

(4) $$\lambda_{1}=\frac{w}{w_{0}}\;\;\;\;\;\;\;\;\;\;\;\;\;\;\;\;\;\;\;\;\;\;\;\lambda_{2}=\frac{h}{h_{0}}\;\;\;\;\;\;\;\;\;\;\;\;\;\;\;\;\;\;\;\;\lambda_{3}=\frac{L}{L_{0}}\;$$

As previously mentioned, Pouillet’s Law can be used to determine the resistivity of a certain material based on its cross-sectional area. The resistance of the channel in our analytical model will be described as:

(5) $$R=\frac{\rho L}{wh}$$

For an incompressible neo-Hookean material under uniaxial tension or compression, the following relationships can be derived:

(6) $$\lambda_{1}=\lambda_{2}=\frac{1}{\sqrt{\lambda_{3}}}\;\;\;\;\;\;\;\;\;\;\;\;\;\;\;\;\;\;\;\;\;\;\;\;\;\;\;\;\;\;\;\;\;\;\;\;\;\;\;\;\;\;\;\;\;\;\;\;\;\;\lambda_{3}=\frac{L}{L_{0}}$$

Solving the system of equations in terms of $\lambda_{3}$, the resistance of the channel can be expressed using the following equation:

(7) $$R=\frac{\rho L_{0}\lambda_{3}{}^{2}}{w_{0}h_{0}}$$

Dividing the resistance by its initial value, a relationship between the length of the channel and the resistance can be derived.

(8) $$\frac{R}{R_{0}}={\left(\frac{L}{L_{0}}\right)}^{2}=\lambda_{3}^{2}$$

This relationship will be used to confirm the validity of the process used to perform the FEA simulations and the corresponding computational results. Once the initial resistance of the static sensor is found, it can be multiplied across an array of length values to determine the final resistances at each length. These final resistance values will be cross-examined with the FEA simulations to determine the degree of agreement between the two models. With good agreement, we will have confidence in further exploring the different modes of deformation as described in Section 2.3.

The force applied to a neo-Hookean material under unilateral compression can be described using the following equation:

(9) $$F=\frac{EA_{0}\left(\lambda_{3}-\lambda_{3}{}^{-2}\right)}{3}$$

Here, E is that elastic modulus of the material and *A*_0_ is the initial surface area in which the force is applied. Using this equation, we can find an analytical relationship between the force and change in lateral length of the sensor. In turn, using the Poisson’s ratio of the material, we can approximate the change in length of the channel with respect to force. Finally, the relationship in Equation (8) can be applied to find an analytical relationship between force applied and resistance of the sensor. This relationship will be compared to the FEA model to further determine the goodness of fit between the two models.

## 3. Results and Discussion

### 3.1. Resistance vs. Force

**Figure 4:**
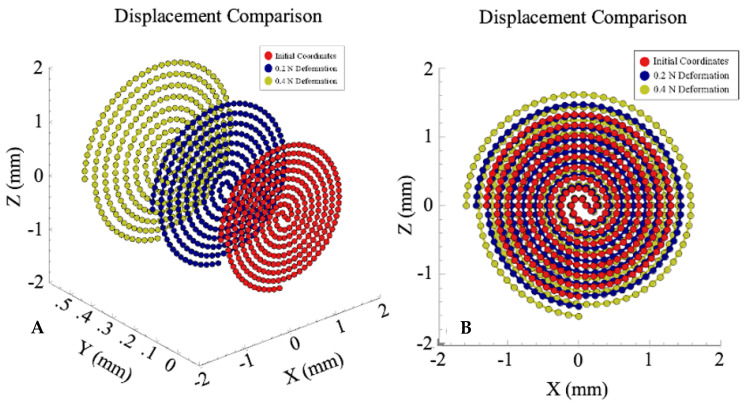
(**A**) The initial and deformation after 0.2 and 0.4 N channel coordinates are plotted above. The channel experienced an average Y displacement of 0.271 mm and 0.542 for the 0.2 and 0.4 N cases, respectively. (**B**) The zx-plane view of (**A**). The change in length of the channel is subtle near the center of the sensor but becomes more pronounced as the channel expands distally. The total length change for the 0.2 and 0.4 N cases is 4.48 and 8.97 mm, respectively.

Using FEA simulations, a parametric sweep of force was applied to the sensor (0–0.4 N, step size of 0.1 N). The change in length and cross-sectional area was calculated using the distance formula and a constant volume assumption. As expected, the length of the channel increased proportionally with the force applied, while the cross-sectional area decreased inversely to force applied. A graphical depiction of channel displacement due to force can be seen in Figure 4. As the force applied increased, the overall displacement of the channel and the channel length increased proportionally. The length of the channel increased by 2.24 mm/0.1 N. The final length for the maximum force value applied (0.4 N) was 46.3 mm, an increase of 8.97 mm. The cross-sectional area for the 0.4 N case decreased by a total of 0.246 mm^2^.

**Figure 5:**
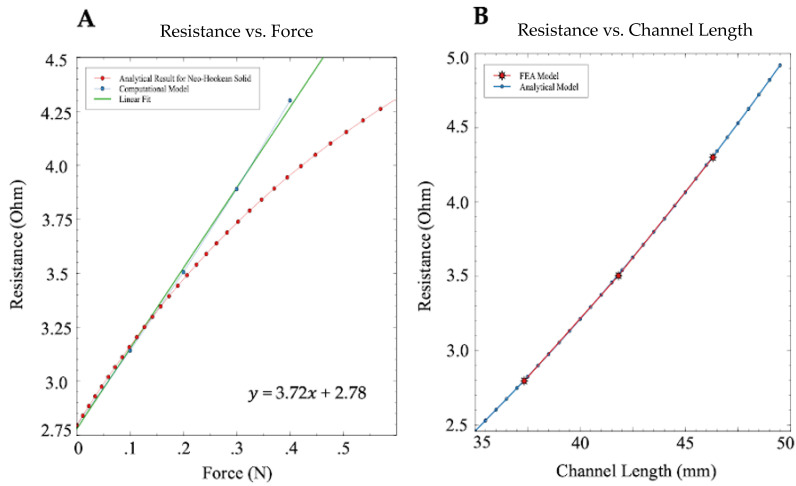
(**A**) Resistance vs. force relationships found through FEA modeling and analytical approaches. Matlab’s curve fitting application was used to create the linear approximation, with the equation of the line displayed on the bottom right-hand side. The range of highest ablation success rate (0.1–0.2 N) displays high agreement between the two models. (**B**) The resistance vs. channel length trends for both the analytical and FEA models are plotted above. The FEA model values are based on applied force values (starting left, going right) of 0, 0.2, and 0.4 N.

The change in resistance for each case was calculated for all line segments that formed the spiral channel using Pouillet’s Law. The final resistance of the sensor was calculated for each case and can be seen in Figure 5, plotted against its respective force value. The maximum resistance increase was 1.5 ohms in the 0.4 N case. Using a linear model, the resistance increased approximately by 0.37 ohms/0.1 N. The analytical solution of the sensor modeled as a neo-Hookean material is also shown in Figure 5A.

A neo-Hookean model was used to analytically determine the sensor’s change in electrical resistance in response to an increasing channel length. Using a model suitable for uniaxial compression of an incompressible solid, a relationship between the resistance after deformation and length of the channel was found (see Equation (8)). Using the initial resistance of the proposed geometry, the theoretical resistances across channel lengths of 35–50 mm were calculated while maintaining a constant initial length of 37.4 mm. 

The resulting resistances and their respective channel lengths from the force sweep in Section 3.1 were overlaid onto the theoretical resistance values previously mentioned above. The resistance vs. length trends for both models can be seen in Figure 5B.

With only a slight deviation due to the analytical model representing the channel as a rectangle rather than a semicircle, the two models yield the same result. This is expected since both models assume constant volume of the liquid metal channel in calculating the total resistance. This result confirms the validity of our FEA modeling method with regard to the conversion of the length of the channel to resistance, while only small errors exist in the conversion from force to change in length after a 0.2 N threshold. The agreement between models allowed us to continue to explore different forms of contact across the sensor utilizing FEA with confidence that the result of the simulations will reflect that of a neo-Hookean material.

Overall, the fitted lines are in reasonable agreement with the computational and analytic models. Pouillet’s Law depicts a linear relationship between the change in length of a channel, and the change in resistance when the change in length greatly outweighs the change in cross-sectional area. The analytical model follows the linearity of the FEA model up to 0.2 N force, beyond which it starts to deviate from the linear elastic model. The deviation can be explained by differences in force vs. length relationships between the two models, as the resistance vs. channel length remains indistinguishable as shown in Figure 5B. However, for the range of forces anticipated in catheter ablation applications (0.1–0.2 N), there is strong agreement between the two models.

### 3.2. FEA Simulation, Increasing Compression Area

**Figure 6:**
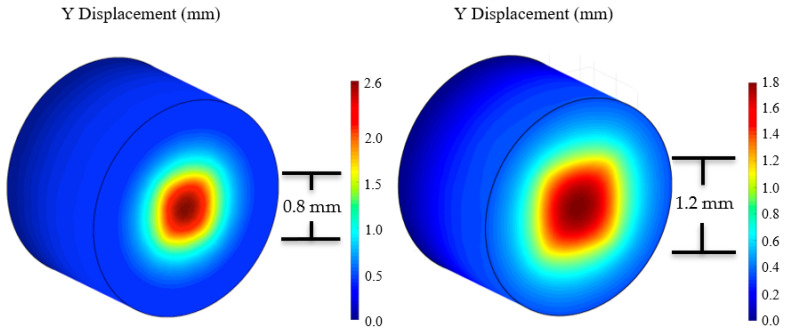
FEA simulation showing increase in the deformation area under compression applied to a central, square area. The figure on the left corresponds to compression with a central square of 0.8 by 0.8 mm in size, whereas the right figure shows compression with a 1.2 by 1.2 mm central square. Each simulation’s data was exported and used to calculate the resistance across the sensor. A parametric sweep of the surface area was simulated starting at a 0.8 by 0.8 mm square area and ending at a 2 by 2 mm square area.

Depending on the size of ablation tip integrated with the sensor, the overall sensor surface area under stress may change. This would affect the overall sensor resistance and output. To account for these potential discrepancies, the resistance as a function of an increasing surface area was determined using the method described in Section 2.3. A central square compression was used to represent the impression of the ablation tip on the sensor. The square compression increased in size to represent different sized tips that may be implemented. A constant 0.4 N force was used for each simulation to ensure the change in resistance was strictly due to the changing area. However, because our model is linearly elastic, this relationship can be used for any force value as long as a multiplier is applied. Figure 6 illustrates two completed simulations with varying surface areas under stress, displaying the trend visually. 

**Figure 7:**
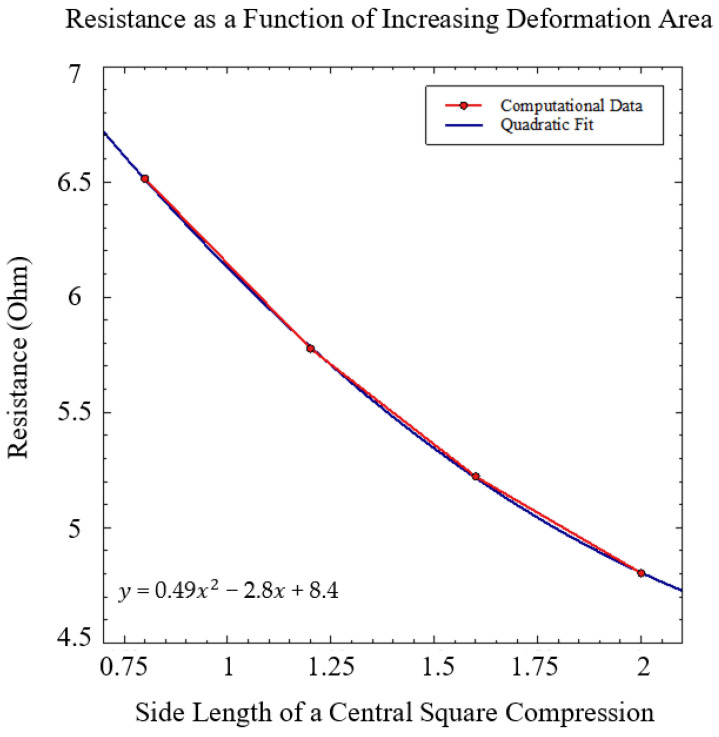
The resistance as a function of expanding deformation area was determined. An expanding square compression (see Figure 6) at a constant 0.4 N force was simulated using a parametric sweep of the square’s side length. A quadratic function was fit to the to the data points, with the equation of the line displayed.

A parametric sweep of the side length was used to determine the change in area for each simulation. A quadratic relationship between the resistance and the expanding area was found and can be seen in Figure 7. As the area increased, the resistance decreased in a quadratic fashion. This relationship is caused by the increased pressure needed to maintain a 0.4 N force in a smaller area. The increased overall pressure in the case of a smaller compression area causes a much larger deformation of the embedded liquid metal channel compared to a more dispersed pressure. This deformation decreases the overall cross-sectional area of the centrally located portion of the channel much more dramatically than for other loading conditions. The cross-sectional area of the channel is located in the denominator of Pouillet’s law (Equation (2)), causing exponential growth of the resistance at miniscule areas.

**Figure 8:**
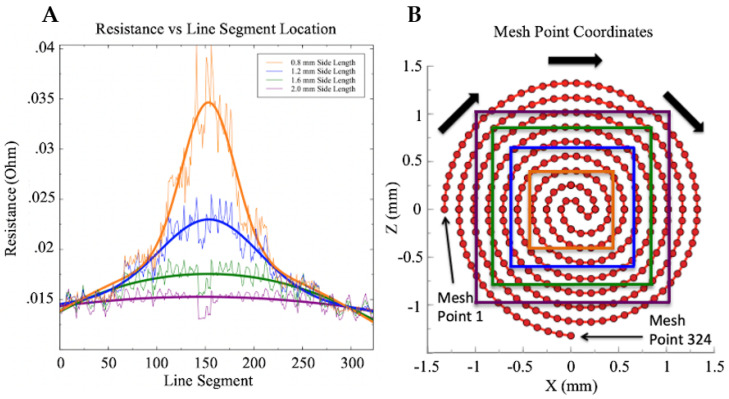
(**A**) Small central compressions displayed an exponential increase in resistance of central line segments. As in Figure 3B, the sensor channel is made up of 324 line segments, with the center being composed of segments 150–170. The figure above displays the resistance generated per segment. As expected, the resistance per segment drastically increased in the center of the sensor when a concentrated pressure was applied. As the central compression area increased, the resistance per segment decreased towards the center of the sensor decreased. (**B**) The above figure represents Figure 3B overlaid with the compression area used in each simulation trial. Each color of the square contacts corresponds to the curves plotted in (**A**).

To further examine this, the resistances of each individual line segment that constitute the channel were plotted against their corresponding location. The leftmost starting point of the spiral structure that can be seen in Figure 8B is classified as point 1 and the beginning of line segment 1. 

Likewise, the bottommost starting point of the spiral structure is classified as point 324, the ending of line segment 323. The center of the channel corresponds to median values of the range 1–324. The results of this plot can be seen in Figure 8 below.

The raw computational data can be seen as sporadic, noisy oscillations, with a bell-shaped fit over each data set. A second order Gaussian was used to fit each data set, with the adjusted R squared value for each fit increasing as the size of the central square compression decreased. The ending condition of 0.8 mm side length displayed a 0.94 adjusted R squared. This plot supports our previous intuition, showing an exponential increase in resistance as the line segments trend toward the center of the sensor. Experimental data recorded on fabricated soft sensors have also shown this exponential resistance trend as pressure is increased [15,17].

### 3.3. FEA Simulation, Lateral Pressure Loading 

**Figure 9:**
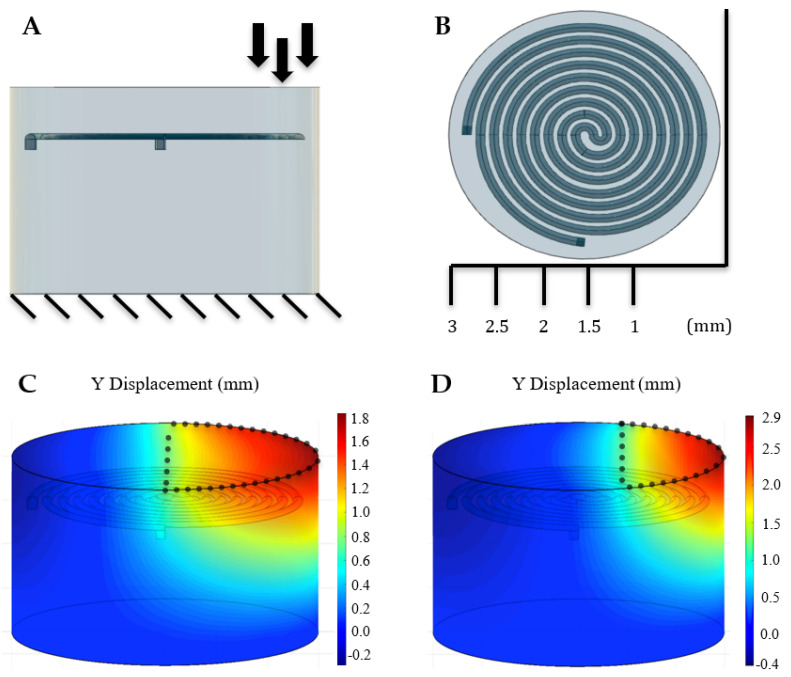
(**A**) A free body diagram displaying the conditions in which the lateral pressure sweep was executed. A constant force of 0.4 N was dispersed between the rightmost boundary of the sensor, and one of the X-positions shown in (**B**). This sweep represents the conditions the sensor would be under if there was an imperfect uniliateral contact of the ablation tip with the heart wall. Having the relationship between resistance and lateral pressure loading would allow adjustments to be made to the output reading if imperfect contact were to occur. (**C**,**D**) The previously mentioned sweep was simulated using Matlab’s FEA tool. Pressure varied between each simulation to maintain a total force of 0.4 N. The above figures display two completed simulations used for this test. The left figure represents pressure applied to the surface area less than 1.5 mm, or half of the entire sensor. The figure on the right corresponds to values less than 1.0 mm. The total area of force exerted is displayed by the black dotted outline.

In cardiac catheter ablation, due to the ex vivo control by the surgeon, it is often hard to create a uniform unilateral contact with the beating heart. As we saw in the previous section, the sensor’s resistance output will be highly dependent on surface area under load. Therefore, we investigated resistance, as a function of an increasing lateral pressure loading to mimic the angled contact the ablation tip would have with our sensor. Such a loading could arise if contact with the heart was not completely unilateral. Using a constant 0.4 N force applied on the sensor, a parametric sweep of x-axis location was used to determine how the resistance would be affected if the force was only distributed in area bounded by that location. Figure 9 displays a free body diagram to illustrate the tests and x locations used along with two completed simulations of this sweep. Similar to Figure 8, this figure displays the trend of the sweep visually.

**Figure 10:**
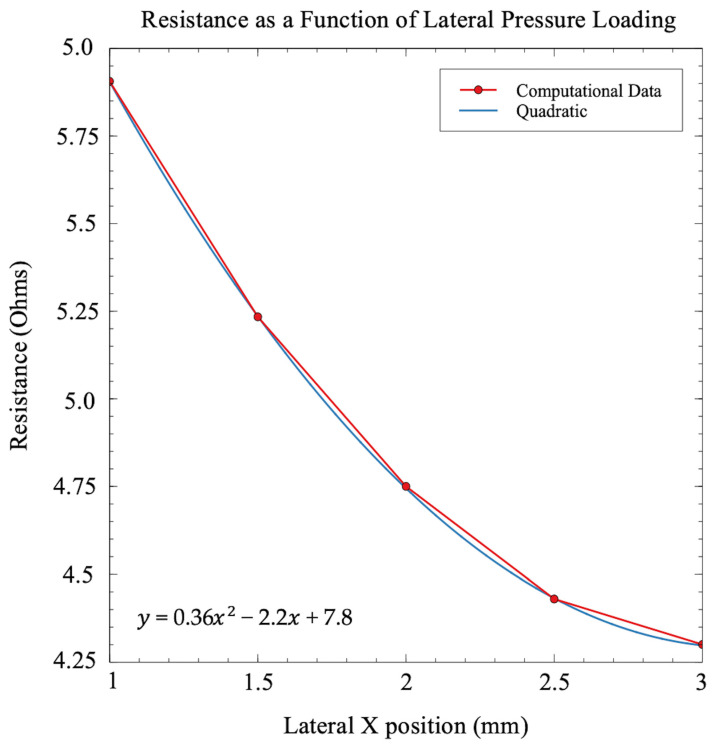
The resistance as a function of lateral pressure loading was determined. A lateral compression defined by Figure 9 was simulated at a constant 0.4 N force. A quadratic function was fit to the to the data points, with the equation of the line displayed.

The parametric sweep of the specified X locations began at X = 3.0 mm, or the entire surface area of the sensor. The sweep ended at X = 1.0 mm, as seen in Figure 9. The results of the resistance as a function of lateral pressure loading can be seen in Figure 10. Similar to the square compression simulations, the resistance forms a quadratic relationship with laterally decreasing compression area. While the overall trend between Figure 7 and Figure 10 is the same, the quadratic fits have slight differences between them. While each figure has a differently defined x-axis, both figures can be looked at as a function of surface area. The specific length of the channel under intense compression can explain the most likely reason for this slight difference in trend. Near the edge of the sensor, the channel length is greater due to larger diameter spiral turns. More channel length under higher compression will lead to a more pronounced exponential trend.

## 4. Discussion and Conclusions

Using FEA, we examined various modes of force loading of a soft resistive elastomeric sensor. The proposed geometry of the sensor and semicircular channel was based off the novel fabrication technique created by Parekh et al. [19] and the spiral design featured in [15]. Our sensor was modeled as an incompressible material and used the inherent properties of EGaIn and EcoFlex 0030 to model our conductive liquid and elastomer respectively. To examine the validity of our FEA simulations, a neo-Hookean analytical model (widely used for elastomeric materials) was implemented under the same conditions and compared to the computational predictions.

Using our FEA model, a linear correlation between resistance and force applied was found when the surface area under load remained constant. This result was expected due to Pouillet’s Law depicting a linear relationship between the length of the channel and resistance when the change in cross-sectional area is negligible. With the cross-sectional area already magnitudes smaller than our channel length, we expected the length to dominate the resistance when a constant volume assumption is made.

A neo-Hookean analytical model was used to compare the change in resistance of the sensor under force with the results from our FEA model. The conversion of resistance to channel length was identical, which was expected as both methods use a constant volume assumption to convert between the two values. When comparing resistance to force applied, the models displayed high agreement up to 0.2 N, which is the recommended force used for cardiac catheter ablation as previously mentioned. After 0.2 N, deviations between the two models start to occur that will need to be compensated for in further studies. However, the overall reasonable agreement gives us confidence that we have an understanding of the sensor’s underlying mechanics.

After initial model validation, different methods of loading were examined computationally. These loading methods were created to mimic the different angles of tissue contact the ablation tip may encounter during the procedure, and account for the different styles of ablation tip impacting the sensor. Similar quadratic relationships between resistance and load were found dependent on the type and placement of the load. The quadratic nature of these trends can be explained by the increased localized pressure needed to maintain a constant 0.4 N force applied.

The next step is to fabricate our proposed design and conduct experimental trials on the sensor. The experimental resistance would be determined under the same conditions illustrated in this article to determine the agreement between the experimental data and our proposed models. Finding an accurate model for a 3D-printed sensor will allow for better fabrication repeatability of the microsensor with the ultimate goal of using the sensor for cardiac catheter ablation. Once successful, design modifications will have to be implemented to integrate the soft actuator and sensor into a function RF ablation catheter tip.

In cardiac catheter ablation, the catheter is hard to control due to the remote, ex vivo control by the surgeon. Along with the constant beating and abnormal surface geometry of the heart, it is difficult to create an exactly uniaxial contact with the heart wall. Therefore, it is crucial to be able to sense the force applied to the heart walls at an angled approach. We found a relationship between the resistance and lateral loading conditions that could be used to model angled ablation. Future research will be needed to create an experimental relationship between angle of contact and the lateral x position that we defined so that a relationship between resistance and angle of contact at a constant force can be created.

While similar designs have been created and experimentally shown to induce a change in resistance under deformation [14,15,27,28], here we focused on a computational study to characterize resistance vs. force relationships for liquid metal sensors under various loading conditions. Park and Majidi et al. displayed a strong relationship between theoretical and experimental results for both centered and off centered forces applied to soft liquid metal sensors but did not include a complete computational study. Gao et al. briefly displayed an experimental change in resistance vs. force relationship that displayed around a 0.4 ohm/0.1 N at small forces, comparable to our computational 0.37 ohm/0.1 N [28]. However, the forces described in Gao et al. were purely tensile, differing from the compressive forces investigated in this work. While the simulations we ran were based on our personal research goals, we hope that these preliminary results can shed light on what to expect for others in this line of work, regardless of application.
